# In Vitro Propagation of Alyssum Species with Different Metal Accumulation Strategies

**DOI:** 10.3390/plants13223122

**Published:** 2024-11-06

**Authors:** Mirosława Górecka, Anna Koszelnik-Leszek, Anna Rusaczonek, Natalia Marek, Oliwia Matz, Ewa Muszyńska

**Affiliations:** 1 Department of Botany, Institute of Biology, Warsaw University of Life Sciences-SGGW, Nowoursynowska 159, Building 37, 02-776 Warsaw, Poland; miroslawa_gorecka1@sggw.edu.pl (M.G.); anna_rusaczonek@sggw.edu.pl (A.R.);; 2 Department of Botany and Plant Ecology, Wrocław University of Environmental and Life Sciences, Norwida 25, 50-375 Wroclaw, Poland; anna.koszelnik-leszek@upwr.edu.pl

**Keywords:** germination, Lepidium test, hydrogen peroxide, sodium nitroprusside, multiplication, metallophytes

## Abstract

The Alyssum genus, with its many metal-adapted species, is a good candidate for research on phytoremediation and metal tolerance mechanisms. These goals can be supported by elaborating on an in vitro multiplication protocol. Our study aimed to determine the aseptic conditions for the growth and effective propagation of *Alyssum murale*, *A. alyssoides*, and *A. montanum*, each exhibiting different adaptation strategies to nickel ions. Firstly, hydrogen peroxide (H_2_O_2_) or sodium nitroprusside (SNP) were investigated in the biological Lepidium test to find their optimal concentrations that could improve the germination attributes of tested Alyssum species. The concentration of 0.5 mM H_2_O_2_ or SNP was selected for research on Alyssum seeds, which were the initial material to start in vitro cultivation. Regardless of the species, H_2_O_2_ harmed germination percentage; however, its application accelerated radicle emergence, especially in metal-sensitive genotypes, while in both metal-tolerant ones, the germination time of H_2_O_2_-treated seeds was similar to that of treated with SNP. These findings provide a novel insight into the effect of H_2_O_2_ or SNP on seeds, contributing to a better understanding of their role in the germination of different genotypes. Among tested media compositions, the synchronous plant regeneration of all species was achieved on MS medium supplemented with 0.5 mg/L 2iP and 0.1 mg/L IAA, making an essential advancement in the in vitro protocols for metallophytes.

## 1. Introduction

Over the last dozen years, plant cell, tissue, and organ cultures have developed dynamically and revolutionized various fields of biology. In vitro techniques have numerous commercial applications and provide benefits for different industries. They facilitate the breeding of plants with improved, changed quality and the production of secondary metabolites or other bioactive compounds with health-promoting properties [1,2]. Furthermore, in vitro cultures play a significant role in the ex-situ conservation of biodiversity through the effective multiplication of rare, endangered, and valuable species before their reintroduction to natural conditions or their long-term storage [3]. Additionally, they are an effective tool for the regeneration of plants uniform in terms of health, genotype, and phenotype, even from an often limited source of material [4]. Micropropagation, as this in vitro vegetative propagation technique is called, is particularly useful if many plants with the same characteristics are needed rapidly and regardless of the growing season [5]. Recently, researchers around the world have been searching for metal-resistant genotypes since constantly increasing environmental pollution with heavy metals is becoming a dangerous global problem. Such plants can be obtained under aseptic conditions by in vitro selection or genetic transformation and their subsequent micropropagation [5,6]. Then, the successfully produced plant material can be directly used for phytoremediation. It is an affordable, eco-friendly, and plant-based biological alternative to traditional methods of purifying metal-contaminated soils [7,8]. In this regard, in vitro cultivation also offers the possibility of large-scale screening of potential candidates for this clean-up technology since it reduces both the space necessary for the tests and the time required for treatments [9]. As an example, *Populus alba* clones [10], *Arundo donax* [11], or *Oryza sativa* [12] were cultured to assess their suitability for two main phytoremediation approaches. The first one, called phytostabilization, includes metal immobilization in the medium or plant roots. Contrary, the second approach—phytoextraction, involves the removal of metal from the surroundings through the active uptake of toxic ions by plant roots and their translocation to shoots [13]. 

Improving the practical possibilities of in vitro utilization for phytoremediation is closely related to fundamental research. In such a case, cell, tissue, and organ cultures are powerful and informative instruments for experiments on the phytotoxicity of metallic elements and plant reactions to these stress factors [14]. The optimized in vitro conditions can be treated as a model system, providing several advantages. Among them, the direct contact of explants with metallic ions added to the medium in precisely defined doses should be mentioned first. Other benefits include the lack of typical soil with its physio-chemical properties that modify ion absorption and the absence of microorganisms influencing metal detoxification [9,15]. In this way, aseptic techniques permit accurate determination of the straightforward effects of tested ions on plants and their defense mechanisms. An innovative approach that combines fundamental and practical research is using in vitro techniques to multiplicate metallophytes, i.e., plants that have been adapted to growth and development in the presence of heavy metals through natural selection [7]. In their case, vegetative propagation under aseptic conditions seems to be particularly justified in obtaining a large amount of plant material, since the generative reproduction of plants growing in natural metalliferous habitats is frequently affected by metals and other environmental stresses [16,17]. Metallophytes show various abilities of metal uptake and translocation within their tissues. Thus, they are classified as excluders, (hyper)accumulators, or indicators. Among them, metal excluders are the largest group, whereas the number of hyperaccumulators is strongly limited only to approximately 720 species from 52 families [18]. Considering the mentioned life strategies in the context of phytoremediation, metal excluders, which retain metals in their underground parts, can be particularly useful for phytostabilization. In contrast, accumulators, especially hyperaccumulators, show an extraordinary ability to accumulate metals in their shoots. These unique plants can be applied for phytoextraction purposes, especially for phytomining, i.e., extraction of metal deposits from soils where they occur in high concentrations but still too low for traditional mining methods to be profitable [13]. The greatest number of hyperaccumulators is found in the Brassicaceae family, and the majority of its representatives are classified in the Alyssum genus [19]. They hyperaccumulate only nickel (Ni) ions, and many of them, such as *Alyssum bertolonii*, *A. lesbiacum*, or *A. murale*, can achieve more than 1000 mg kg^−1^ Ni in dry leaves [20,21]. Other Brassicaceae members have been reported as hyperaccumulators of more than one element, e.g., *Arabis paniculata* is a well-known hyperaccumulator of zinc, lead, and cadmium [22], while *A. gemmifera* hyperaccumulates zinc and cadmium ions [23]. Nevertheless, the Alyssum genus is particularly fascinating, because it also contains metal excluders, such as *A. alyssoides* or *A. montanum* [19,24,25]. It makes the Alyssum genus an ideal model for comparative experiments on metal tolerance and its uptake. The study of different Alyssum species with contrasting metal-accumulation strategies can provide detailed information on the evolutionary mechanisms of plant adaptation to extreme concentrations of toxic elements. A better understanding of these reactions could enable the development of genetically modified plants with enhanced abilities to tolerate, detoxify, and accumulate heavy metals, making them more effective in various soil remediation techniques. Additionally, it may open possibilities for engineering crop species with improved resistance to metal toxicity, ensuring safer food production in polluted areas. On this matter, the prerequisite step for genetic modifications is the elaboration of in vitro procedures for the micropropagation of valuable plant species. 

Our study aimed to optimize in vitro conditions for the cultivation and multiplication of species from the Alyssum genus with different adaptive strategies to metals. For the first time, an effort was made to develop a protocol for the synchronous production of two contrasting metal-tolerant genotypes: *Alyssum murale* (a well-known Ni hyperaccumulator) and *A. alyssoides* (a Ni excluder) and one metal-sensitive: *A. montanum.* The hints for standardized and efficient in vitro multiplication of metallophytes create an opportunity to conduct future basic and applied research on the relationships between environment, metals, and plants. Since we used seeds to initiate aseptic growth, we also checked whether the previously determined concentrations of reactive oxygen and nitrogen species, applied in the form of hydrogen peroxide (H_2_O_2_) and sodium nitroprusside (SNP, a nitric oxide donor), influenced germination attributes of Alyssum species. Such an investigation can benefit the latest experiments on different priming strategies understood as exogenous pre-treatment of plants leading to the induction of more effective and faster defense reactions to successively acting stress factors [26]. 

## 2. Results

### 2.1. Biological Assay with Lepidium Seeds to Optimise Hydrogen Peroxide and Sodium Nitroprusside Doses

Our study used the Lepidium test to analyze the potential effects of different concentrations of reactive oxygen and nitrogen species on seed germination to find doses of H_2_O_2_ and SNP that will not negatively affect germination parameters. The results of the tested parameters are presented in Table 1. 

The germination percentage (GP) reached the lowest value for seeds treated with the highest dose of H_2_O_2_ (Table 1). Similarly, the lowest concentration of this compound ne-gatively influenced GP, which was about 6% lower than in the case of control seeds. The GP of seeds treated with 0.5 mM H_2_O_2_ was comparable to the control. No significant difference in GP was noted between SNP and water (control) treatments, regardless of the applied SNP concentrations. The seed priming with reactive species was beneficial for a-verage radicle length (ARL), which was significantly higher in almost all treatments than the control one. The exception was 0.1 mM H_2_O_2_-treated seeds, where ARL did not differ from the water-soaked seeds. The radicles of 0.5 mM-treated seeds, both H_2_O_2_ and SNP, achieved a similar length of 8 mm. This value was also the highest among tested doses and about two times higher than in the control. 

Relative germination percentage (RGP) and relative radicle growth (RRG) present the tested parameters as a percentage of the control (100%). RGP of Lepidium seeds primed with 0.5 mM H_2_O_2_ or with SNP (regardless of the dose) was comparable to the control. It reached the lowest values of about 94% and 80% for 0.1 mM and 1.0 mM H_2_O_2_-treaded seeds, respectively. The application of H_2_O_2_ doses higher than 0.1 mM and all doses of SNP significantly promoted radicle emergence (RRG) and germination index (GI) by at least 20% of the water-treated seeds, achieving the highest values for 0.5 mM. 

### 2.2. Effects of Priming Treatments on Seed Germination of Alyssum Species

The dose of H_2_O_2_ and SNP that did not interfere with or even stimulate the germination of Lepidium seeds was 0.5 mM. Therefore, it was applied to the Alyssum species seeds in the following experimental step.

*A. murale* seeds were characterized by deficient germination ranging from 26 to 40%, regardless of the treatment (Table 2). The use of H_2_O_2_ or SNP did not improve the GP value, which, in the case of H_2_O_2_-treaded seeds, was the lowest among tested species and was lower by about 15% than in control *A. murale* seeds. Despite this, tested doses significantly accelerated the germination time from more than 11 days to only about 4.5. 

The germination capacity of the second metal-tolerant species—*A. alyssoides*, was the highest compared to other examined species, and its GP reached the value of 97 and 93% for water- and SNP-treated seeds, respectively (Table 2). Similarly to *A. murale*, the application of H_2_O_2_ significantly reduced GP, but in *A. alyssoides* the decrease in the value of this parameter was higher than 50%. Both treatments with reactive species increased seed vigor expressed by the Pieper’s coefficient (PC) in the same way. Consequently, root emergence (germination) was noticed more than one day earlier than in water-treated seeds. 

Non-metal tolerant *A. montanum* seeds germinated the best after soaking in 0.5 mM SNP. In this case, GP was significantly higher, about 10% and 73%, than in control and H_2_O_2_-treated seeds, respectively (Table 2). SNP also improved germination time (PC) from almost 7 days indicated for control seeds to four days calculated for seeds primed in SNP. The fastest seed germination of about one day was noticed for H_2_O_2_ treatment; however, its application simultaneously limited GP the most.

**Figure 1:**
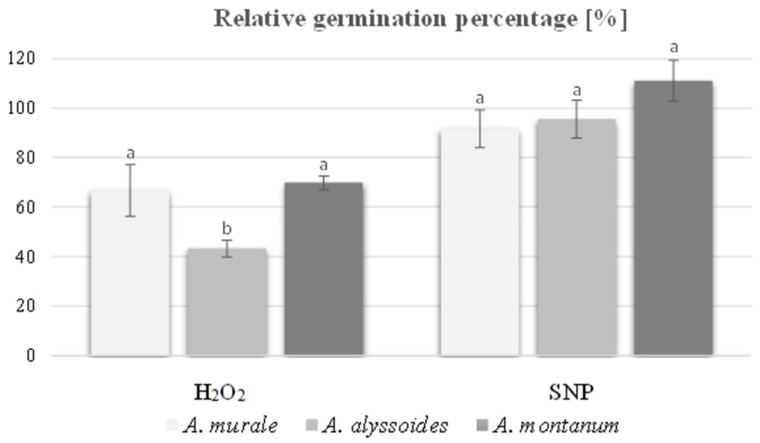
Relative germination percentage of seeds belonging to different species of Alyssum genus under the exposure of 0.5 mM hydrogen peroxide (H_2_O_2_) or 0.5 mM sodium nitroprusside (SNP). Different letters indicate means ± SD that are significantly different at α = 0.05 within a particular treatment according to one-way ANOVA and post hoc Fisher’s test.

As for Lepidium seeds, RGP was also determined for Alyssum seeds. Regardless of the tested species, seed exposure to H_2_O_2_ strongly influenced germination capacity in relation to control treatment (Figure 1). RGP for *A. murale* and *A. montanum* seeds soaked in H_2_O_2_ was similar and its value reached about 70% of the control, whereas for *A. alyssoides* it achieved only 40%. In turn, priming of Alyssum seeds with SNP did not negatively impact RGP, or it provided a slight stimulation for this process in *A. montanum*. 

### 2.3. Evaluation of Culture Parameters Combined with the Selection of the Optimal Growth Medium for Metal-Tolerant Genotypes

Proliferated shoot cultures capable of spontaneous root regeneration were obtained regardless of the medium composition and Alyssum species (Table 3, Table 4 and Table 5, Figure S1). The micropropagation coefficient (MC) of *A. murale* ranged between 1.6 and 4.8 (Table 3). The lowest value of MC was calculated for A1 and A2 media, on which the average number of regenerated shoots from one explant was about 1.65. It was almost three times lower than the highest obtained MC value. The greatest efficiency of shoot multiplication was obtained on the A6 medium (MC = 4.8). However, such intensive formation of new shoots resulted in the shortening of their length, which did not exceed 15 mm. Contrary, the longest shoots, which reached approximately 40 mm, were observed on A1 and A5 media. In turn, the A4 medium was the best for spontaneous rhizogenesis. In this case, 95% of shoots formed adventitious roots with the highest intensity of more than 30 roots per explant. The rate and intensity of root regeneration were the weakest on the medium A1 and A6 together with A3, and the percentage of rooted explants was about 50% and 25%, respectively. Considering all tested features, with scores given depending on their intensity, the most convenient medium for shoot multiplication and spontaneous root regeneration of *A. murale* was the A4 medium (Figure S1). Nevertheless, the medium marked as A5 obtained a similar number of total scores, and the main differences with the A4 medium were related to shoot length and slightly limited rhizogenesis. 

The efficiency of *A. alyssoides* micropropagation was most intensive on the A4 medium (MC = 5; Table 4). In contrast, the shoot proliferation on media A2, A3, and A5 was significantly inhibited, and MC did not reach 3. The length of newly regenerated shoots ranged between 12 and 18 mm, showing the highest value for A1 and A3 media, the lowest—for A2 and A6, and an intermediary value of about 14 mm for A4 and A5. Considering the influence of tested media compositions on root regeneration, it was found that media A3 and A4 turned out to be the best solution for this process, and more than 70% of explants maintained on them produced adventitious roots. The value of this parameter corresponded to the abundance of root formation in the case of the A4 medium (more than 30 roots/explant). In turn, rooting intensity on the A3 medium was comparable to A6 and varied between 10 and 30. Although shoots growing on A1, A2, and A5 media produced at least roots, only in the case of A5 medium the number of rooted explants was not satisfactory, achieving 34%. Taking into account the values of all evaluated growth parameters together, it can be assumed that the composition of the A4 medium led to the complete regeneration of *A. alyssoides* plants with high efficiency of the shoot and root development (Figure S1). 

### 2.4. Effects of Different Medium Compositions on Non-Metal Tolerant Alyssum Montanum Growth

Multiplication of *A. montanum* shoots was the most intensive on the A5 medium (MC = 4.3), while the lowest MC value reaching approximately 1.4 was noticed for the A2, A3, and A4 media (Table 5). The average multiplication was observed in shoots growing on A1 or A6 media. The number of regenerated shoots per explant for these cultures was about three. Shoots on the A1 medium reached the highest length of 33 mm and were about three times longer than the shortest ones from the A3 medium. However, the shoot length similar to these on the A3 medium was also ascertained for shoots formed on A2, A5, and A6 media. Although the best medium for spontaneous rhizogenesis was A6 (85%), the percentage of rooted explants on three media named A1, A2, and A4 was close to 70%. In turn, the growth of new roots on the A5 medium was almost totally inhibited, and rooting characteristics, i.e., the percentage of rooted explants and root number per explant, reached the lowest values of 15% and less than 10, respectively (Figure S1). Contrarily, the most abundant adventitious roots were formed on A4 and A6 media. These last two media proved to be equally valuable for the multiplication of *A. montanum* culture, receiving the highest scores for the parameter tested. A similar number of points (9) was assigned to the A1 medium, which can also be considered suitable for the culture growth of this species.

## 3. Discussion

Recently, H_2_O_2_ and nitric oxide (NO) have become essential compounds in the regulation of various cellular pathways, leading to the seed release from dormancy as well as to the proper growth and development of both seedlings and mature specimens [27,28,29]. What is more, the latest data have shown that the mentioned molecules mitigate the deleterious effects of environmental stresses, even when applied in the pre-sowing stage. It was found that such seed priming increases tolerance to salinity in wheat [30], water deficit in cotton [31], or metal excess in pink trumpet trees [32]. However, the literature reports diversified doses of H_2_O_2_ or NO that can confer positive effects on seed germination and further plant growth under both optimal and adverse conditions. Thus, in the first experimental step, we used the Lepidium test to determine exogenously applied H_2_O_2_ and SNP concentrations that can improve germination parameters. This quick and simple bioassay is widely utilized in biological and environmental studies due to its easy handling, non-intrusive root growth observations, and low cost [33]. Importantly, cress belongs to the same Brassicaceae family as Alyssum, so we assumed the results obtained using this method would be much more comparable and reliable. Our study revealed that reactive oxygen or nitrogen molecules promoted contrasting responses in *Lepidium sativum*. It was found that the application of SNP had no effect on the germination capacity compared to water-treated Lepidium seeds. Still, it increased relative radicle growth (RRG) and germination index (GI), which takes into account not only germinated seeds but also root emergence [33]. The highest values of RRG and GI (up to 180% of the control) were noticed for the 0.5 mM SNP, suggesting the stimulation of seedling growth processes. Indeed, exo-genously applied NO is well known to break seed dormancy and accelerate early plant development in numerous species, such as *Arabidopsis thaliana* and *Hordeum vulgare* [34] or *Brassica napus* varieties [35]. On the contrary, H_2_O_2_ at the concentration of 0.1 or 1.0 mM harmed Lepidium germination. In comparison, 0.5 mM H_2_O_2_ did not change germination capacity, and it was simultaneously beneficial for RRG and GI, reaching about 190%. According to the concept of ‘oxidative window for germination’ introduced by [36], which describes the dual role of reactive oxygen species (ROS) in seed physiology, only their balanced level can successfully initiate the germination, while levels below or above the critical range of the oxidative window impair this process. Therefore, the germination percentage of Lepidium seeds varied among H_2_O_2_ treatments, and the lower H_2_O_2_ dose likely helped maintain dormancy. In turn, the higher dose could induce oxidative stress that accelerates radicle growth compared to the control and promotes seed senescence [37]. Based on these findings, a concentration of 0.5 mM of H_2_O_2_ and SNP was selected for the subsequent step of our experiment on three Alyssum species representing different tolerance levels to Ni ions. 

The tested seeds of Alyssum were taken from natural habitats, so they germinated weaker than the commercially available cress seeds. Nevertheless, they responded similarly to each other. Regardless of the species, the concentration of H_2_O_2_ used significantly reduced the germination capacity. However, the most potent inhibitory effect on relative germination percentage was found in *A. alyssoides*. Considering that this species has evolved in areas heavily polluted with Ni, the excess of which triggers the overproduction of ROS, our result may suggest a high conservative level of ROS in its organs, including seeds. Therefore, the application of an additional H_2_O_2_ dose, which was supposed to increase germination, produced the opposite effects, probably resulting from the disturbance of the intracellular oxidative balance. Despite the negative influence of H_2_O_2_ on the percentage of germinated seeds, the treatment with this molecule shortened the time required for radicle emergence of all species. The most pronounced change in this parameter was noticed for seeds of metal-sensitive *A. montanum*, for which the emergence rate was accelerated by almost seven times compared to water-imbibed seeds. A similar result was observed by [38] for seeds of *Pisum sativum* or by [29] in *Beta vulgaris*, who proposed that the stimulation of germination can be linked to the selective oxidation of various seed proteins and transcriptome remodeling by H_2_O_2_. In turn, priming of Alyssum seeds with SNP provided effective germination at a level analogous to that of water-soaked seeds in both metal-tolerant species and even significantly higher in the non-metal tolerant genotype. Additionally, SNP-treated seeds germinated faster than control ones, and the ave-rage number of days from sowing to germination, expressed by Pieper’s coefficient, was significantly reduced. Interestingly, the germination speed of both SNP and H_2_O_2_-primed seeds was comparable in the case of *A. murale* and *A. alyssoides*, and it differed for *A. montanum* with a predominance of H_2_O_2_. Undoubtedly, both reactive oxygen and nitrogen species contribute to the coordination of life processes in the Alyssum genus, but their mode of action in metallophytes is rarely investigated. The findings of [21] suggested that NO can act as a signaling molecule in Ni hyperaccumulator—*Alyssum lesbiacum*, and its tolerance is related to the balance of reactive nitrogen species and the ability to prevent the proteome from nitration. On the other hand, our previous experiments on the metal-tolerant ecotype of *Silene vulgaris* revealed that shoot priming with NO or H_2_O_2_ caused the alternation in plant morphology accompanied by DNA and protein oxidation [39]. It was also found that Zn-tolerant *Handroanthus heptaphyllus* seedlings treated with NO exhibited higher metal absorption, whereas the application of H_2_O_2_ induced avoidance strategy [32]. Summarizing, the response of metallophytes to reactive oxygen and nitrogen species is probably dose- and molecule-dependent, and the intracellular balance of H_2_O_2_ and NO in these plants can be easily disturbed. For this reason, future detailed studies are needed to understand better these active compounds’ role in metal tolerance and ion accumulation. The broadening of knowledge in this field would also be valuable for improving the phytoremediation and revegetation techniques of areas strongly contaminated with metals. 

Effectively germinating seeds are an excellent source of material for the initiation of in vitro culture aimed at the mass vegetative propagation of species exhibiting unique features. Such an approach was previously reported for different metallophytes like *Alyssum corsicum* [40], *Gypsophila fastigiata* [5], or *Viola arvensis* [6]. Similarly, seeds of *Alyssum murale, A. alyssoides,* and *A. montanum* were used to determine the composition of the growth medium for successful plant regeneration and multiplication. The optimization of microplant production under controlled conditions may contribute to resolving the ever-increasing problem of environmental metal accumulation in a sustainable way [41]. An ecologically safe solution in this respect may be assisted by revegetation of degraded terrains using local, well-adapted to the prevailing conditions metallophytes, which will not threaten native biodiversity [8]. Nevertheless, among the vast existing literature about metal adaptations, aseptic regeneration of metal-tolerant species is still poorly elaborated and refers only to several species like *Gypsophila fastigiata* [5], *Pteris vittata* [42] or *Noccaea caerulescens* [43]. Moreover, successful micropropagation of metallophytes was achieved by developing callus in the case of *Arabidopsis halleri* [44], *Silene vulgaris* [45], or *Sedum alfredii* [46]. Thanks to our research, the pool of metallophytes propagated in vitro has been expanded by the Alyssum genus, whose representatives can be used in contrasting phytoremediation techniques due to their different metal accumulation strategies. Nevertheless, our experiment is based on direct organogenesis, which reduces the risk of genetic variability in regenerants. A similar approach was used by [47], who developed a micropropagation protocol bypassing the callus stage for *Dianthus cruenthus* from serpentinite habitats for future revegetation of metal-polluted sites. Regardless of Alyssum species, MS basal salts and vitamins gave better results than WPM ones for establishing shoot culture. Generally, WPM medium is dedicated to woody plant micropropagation, and it contains low total nutrient amounts and high levels of sulfate and magnesium ions [48]. However, it was previously successfully applied for our experiments with contrasting ecotypes of *A. montanum* (e.g., [25,49]). It is consistent with the current findings on non-metal tolerant specimens, for which the application of A1 medium based on WPM but enriched additionally with activated charcoal as compared to other WPM bases (A2 and A3) supported shoot growth. Consequently, the multiplication coefficient and shoot length were similar to that observed on the best MS media for this species, described as A4 and A6. The WPM medium was also expected to improve the culture growth of both *A. murale* and *A. alyssoides* since these metallophytes spontaneously occur on ultramafic soils characterized by nutrient deficiency [24]. Surprisingly, the most intensively proliferated and spontaneously rooted shoots of these species were obtained on MS medium with a high nitrate and potassium content, enriched with 0.5 mg/L 2iP and 0.1 mg/L IAA (described earlier as A4). The same medium composition but containing two times higher doses of plant growth regulators than tested in the present study also stimulated the regeneration of *Dianthus carthusianorum* specimens cultivated for the phytostabilization of post-industrial wastes polluted with zinc, lead, and cadmium [41]. Apart from A4 medium, MS medium supplemented with BAP and NAA can be helpful for Alyssum micropropagation. However, the applied concentration of this synthetic cytokinin and auxin should be similar for both metal-tolerant genotypes and two times higher for metal-sensitive ones. Based on the current findings, it can also be seen that *A. alyssoides*, compared to other tested species, showed particular preferences for the growth on media enriched with calcium gluconate. It probably results from its natural habitats, including limestone soils [50]. 

The optimized micropropagation protocol for Alyssum species leads to the regeneration of completed plants because the shoot branching was accompanied by the induction of spontaneous rhizogenesis. Typically, the rooting process requires an additional cultivation step as observed for other micropropagated metallophytes like *Dianthus cruenthus* [47] or *D. carthusianorum* [41]. As a result of the applied experimental scheme, our innovative approach provides the shortened time required for the mass production of plants for practical application. In turn, elaborating on the one universal and standard medium composition for several species, like the medium A4 for the Alyssum genus, seems crucial for comparative basic research. Then, the straightforward effect(s) of tested, potentially stressful factors can be assessed more accurately by excluding the possible impact of the medium’s compounds on the plant reactions. So far, in vitro techniques have been used to examine the responses of different metal-tolerant ecotypes of the same species to metal exposure, like in the case of *Silene vulgaris* [51] or *Armeria maritima subsp. elongata* [52], representing geographically isolated populations from metal-polluted sites. Additionally, in vitro cultivation has often been applied to investigate the influence of different metals on one metal-adapted species, such as *Gypsophila fastigiata* [5], *Viola arvensis* [6] or *Sesuvium portulacastrum* [53]. In the present study, the uniform medium has been developed for the first time to support multiple representatives of the same genus with distinct metal tolerance and accumulation traits.

## 4. Materials and Methods

### 4.1. Biological Test for Determination of Optimal Concentration of Hydrogen Peroxide and Sodium Nitroprusside

A Lepidium test was carried out to determine the amount of H_2_O_2_ and SNP doses that could improve seed germination. Briefly, the test used garden cress seeds (*Lepidium sativum*) obtained from a commercial supplier in Poland (Legutko Company, Jutrosin, Poland), which were soaked for 24 h (in the dark and room temperature) in an aqueous solution of H_2_O_2_ or SNP at the concentration of 0.1, 0.5 or 1.0 mM. Control treatment consisted of seeds primed with demineralized water. Then, seeds were placed into the Petri dishes lined with three pieces of filter paper, and 5 mL of demineralized water was added. Four replicates (dishes) of 50 seeds per treatment were applied. The Petri dishes were co-vered and maintained in the dark at 24 °C ± 2 °C for 24 h. After this time, the number of germinated seeds was counted, and the length of 15 radicles per replication showing the best growth was measured. The following calculations [33,54] were made to evaluate the effects of tested concentrations of H_2_O_2_ or SNP on:

(1) seed germination:

Germination Percentage (GP) = (number of germinated seeds in the final day/number of tested seeds) × 100%,

Relative Germination Percentage (RGP) = (number of germinated seeds treated with H_2_O_2_ or SNP/number of germinated seeds treated with water) × 100%,

Relative Radicle Growth (RRG) = radicle length of the germinated seeds with H_2_O_2_ or SNP/radicle length of the germinated seeds with water) × 100%.

(2) seed germination and root growth simultaneously:

Germination Index (GI) = (RGP × RRG)/100%.

### 4.2. Estimation of the Primed Seeds’ Vigor in the Alyssum Genus

In the main experiment, seed samples of three Alyssum species with different adaptive strategies to nickel ions were tested:

(i) *A. murale* (Waldst. et Kit) ‘Kotodesh’, a Ni-hyperaccumulator,
(ii) *A. alyssoides* (L.), Ni-excluder,
(iii) *A. montanum* (L.), a non-metal tolerant genotype.

Seeds were treated for 24 h with H_2_O_2_ or SNP in the concentration determined during the previously described Lepidium test. The dose of 0.5 mM H_2_O_2_ or SNP was selected for this research. Demineralized water-treated seeds were used as a control. Three hundred seeds of every species were applied (one hundred per treatment). Then, the seeds were surface decontaminated in 70% (*v*/*v*) ethanol for 1 min and in 0.05% mercuric chloride for 4 min. The duration of treatment with mercuric chloride was chosen based on the preliminary experiment when times from 1 to 7 min were tested. After five washes with sterile water, they were placed in Petri dishes onto MS medium [55] with macro- and microelements reduced to half, without vitamins and plant growth regulators. Ten seeds per plate were put and maintained in an air-conditioned growth chamber (MLR-350, Sanyo, Tokyo, Japan) at a temperature of 24 °C ± 2 °C, under white light with a quantum radiation intensity of approximately 80 μmol m^−2^ s^−1^, with a photoperiod of 16/8 h. Newly germinated seeds (i.e., those having at least 2 mm long radicle protruding through the seed coat) were counted until their number remained constant (i.e., no longer than 21 days after sawing) every day at the same time. Finally, GP and RGP were calculated for each treatment and species. Additionally, the Pieper’s coefficient (PC) showing the mean germination time (days) was also rated using the formula [56]:

Pieper’s Coefficient (PC) = ∑(number of the germination day × number of seeds germinated on a given day)/∑ number of seeds germinated on a given day

A low calculated PC value indicates fast germination and high seed vigor, while a high value suggests extended time from sowing to root emergence (germination) and, thus, low seed vigor.

#### Experimental Scheme for Micropropagation

**Figure 2:**
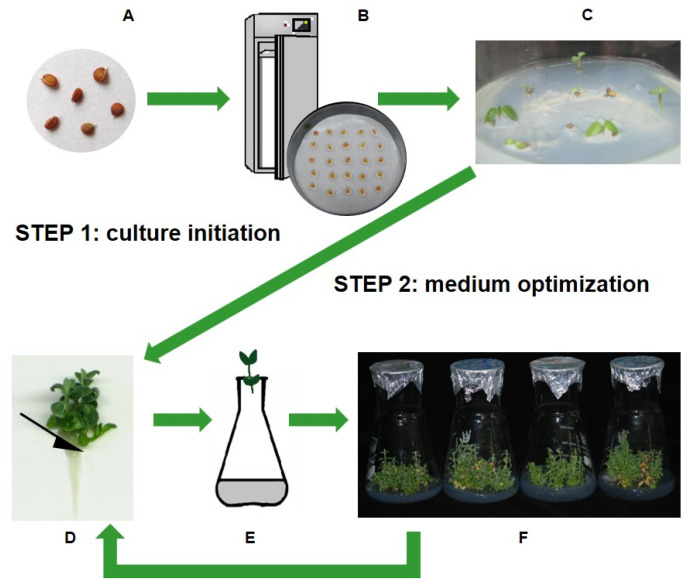
The subsequent stages of the experiment on developing the optimal medium composition for micropropagation of various species from the Alyssum genus: (**A**)—seeds as an initial explant for in vitro cultivation, (**B**,**C**)–aseptic seeds germinated on half-strength MS medium without vitamins and plant growth regulators, (**D**)—preparation of explants by cutting the roots from the shoots, (**E**)—putting explants onto freshly prepared medium, (**F**)—culture growth on media with different compositions. The medium optimization step lasted 12 weeks, and every four weeks, newly prepared explants (apical fragments of regenerated shoots) were transferred to fresh media of the same composition. During the experiment, cultures were kept in a growth chamber (at 24 °C day/22 °C night, with an irradiance of 80 μmol m^−2^ s^−1^, with a photoperiod of 16/8 h.

The protocol that enables standardizing in vitro shoot culture for further applied and fundamental studies was elaborated in two steps, as shown in Figure 2. The experimental scheme included the establishment of the aseptic culture from the seeds (Figure 2A–C) before the selection of the best propagation medium (Figure 2D–F). 

Seeds were subjected to surface decontamination (in 70% (*v*/*v*) ethanol for 1 min and in 0.05% mercuric chloride for 4 min) and maintained in the conditions described above for the test of seed germination ability and vigor (Figure 2A,B). Apical fragments of aseptically obtained seedlings (Figure 2C) deprived of roots were used as primary explants to optimize the shoot multiplication protocol (Figure 2D–F).

The tested modifications of media based on the Woody Plant Medium [57] or MS [55] salts and vitamins enriched with different concentrations of organic additives and plant growth regulators, as shown in Table 6. 

Each media was supplemented by 20 g/L sucrose and solidified with 0.9% Difco Bacto agar, and their pH was adjusted to 5.8 before autoclaving (121 °C for 20 min). Vessels of 250 mL capacity filled with 50 mL of the respective medium were used for this experimental step. Eight explants of particular species were put in a single vessel onto a freshly prepared medium, and ten vessels per treatment were maintained. Every four weeks, the two-node fragments of regenerated shoots were transferred to fresh media with the same composition. The entire experiment lasted 12 weeks. Cultures were kept in a growth chamber MLR-350 (Sanyo, Tokyo, Japan) at 24 °C day/22 °C night, under a 16 h photoperiod, and the irradiance of 80 μmol m^−2^ s^−1^. 

### 4.3. Growth Evaluation and Choosing the Optimal Medium for Shoot Multiplication

The biometric measurements were made after 12 weeks of culture. The number and length of multiplicated shoots were ascertained, and the micropropagation coefficient (MC) was calculated as the number of newly regenerated adventitious shoots per total number of explants. The spontaneous root formation (if it occurred) was marked with pluses depending on the rhizogenesis intensity, where “+” meant spontaneous regeneration of up to 10 roots per explant, “++” meant more than 10 but less than 30 roots, and “+++” meant more than 30 roots. 

The obtained data were subjected to statistical calculations, and the optimal medium for micropropagation was determined by scoring the evaluated characteristics based on their intensity. The lowest natural number was assigned to the statistically homogenous groups with the lowest intensity of a given characteristic, while the highest—to the highest intensity. If statistical groups overlapped, the characteristic received the average score of both groups. The number of points given for the assessed characteristics was then summed up for each medium, and the medium with the highest overall score was identified as the best growth medium for a particular species. 

### 4.4. Statistical Calculations

Data were subjected to one-way ANOVA analysis (STATISTICA ver.13.3, TIBCO Software Inc., Palo Alto, CA, USA), and the post-hoc Fisher’s test was used to compare the means either between all doses of H_2_O_2_ and SNP for Lepidium seeds, or between treatments within the same species of Alyssum at α = 0.05. The exception was RGP for Alyssum seeds, where means between different species were analyzed. Tukey’s test for different N was applied for the statistical interpretation of multiplicated shoot length since explants regenerated an unequal number of shoots, and all of them were evaluated.

## 5. Conclusions

The present study revealed that seeds are a good source of plant material to start in vitro cultivation of particular representatives of the Alyssum genus; however, their germination parameters should be increased. In this regard, the application of SNP at the concentration of 0.5 mM proved to be beneficial for the quick and successful establishment of seedlings. Further in vitro cultivation and clonal shoot propagation accompanied by spontaneous root regeneration should be conducted on the standardized medium for all tested species, containing MS micro-, macro-elements, and vitamins enriched with organics (500 mg/L polyvinylpyrrolidone, 650 mg/L calcium gluconate) and phytohormones such as 0.5 mg/L 2iP and 0.1 mg/L IAA. The proposed in vitro protocol provides valuable hints for the cost-effective and time-saving production of large quantities of true-to-type and high-quality microplants with different metal-accumulation strategies. Such multiplicated Alyssum specimens can then be helpful for the remediation of degraded areas, phytomining of Ni ions, and conducting primary research on comparative plant reactions and adaptations to metals under uniform, fully controlled conditions.

## Figures and Tables

**Table 1:** Germination percentage (GP), average radical length (ARL), relative germination percentage (RGP), relative radicle growth (RRG), and germination index (GI) in Lepidium seeds exposed to various concentrations of hydrogen peroxide (H_2_O_2_) or sodium nitroprusside (SNP) after 24 h.

	GP [%]	ARL [mm]	RGP [%]	RRG [%]	GI [%]
water—control	93.00 ± 3 a *	4.36 ± 0.87 d	100.00 a	100.00 d	100.00 e
0.1 mM H_2_O_2_	87.00 ± 5 b	4.24 ± 1.55 d	93.55 ± 6.25 b	97.35 ± 1.96 d	91.14 ± 6.31 e
0.5 mM H_2_O_2_	94.00 ± 2 a	8.18 ± 1.34 a	101.08 ± 4.11 a	187.88 ± 1.61 a	190.03 ± 6.93 a
1.0 mM H_2_O_2_	74.50 ± 4 c	6.42 ± 1.56 b	80.11 ± 4.02 c	146.76 ± 7.27 b	117.72 ± 10.28 d
0.1 mM SNP	90.00 ± 2 a	5.29 ± 1.14 c	96.77 ± 2.68 ab	121.25 ± 3.03 c	117.41 ± 4.61 d
0.5 mM SNP	91.00 ± 1 a	7.91 ± 1.33 a	97.85 ± 1.70 ab	181.61 ± 4.67 a	177.82 ± 6.79 b
1.0 mM SNP	92.00 ± 2 a	6.76 ± 1.11 b	98.75 ± 4.39 ab	153.83 ± 8.39 b	151.71 ± 4.51 c

* Different letters indicate means ± SD that are significantly different at α = 0.05 according to one-way ANOVA and post hoc Fisher’s test.

**Table 2:** Germination percentage (GP) and Pieper’s coefficient (PC) in seeds of different representatives of the Alyssum genus exposed to hydrogen peroxide (H_2_O_2_) or sodium nitroprusside (SNP) in the concentration of 0.5 mM.

	Water—Control	0.5 mM H_2_O_2_	0.5 mM SNP
*A. murale*			
GP [%]	40.67 ± 1.15 a *	26.67 ± 5.77 b	36.67 ± 5.77 a
PC [day]	11.17 ± 0.88 a	4.44 ± 0.51 b	4.58 ± 0.38 b
*A. alyssoides*			
GP [%]	97.67 ± 4.04 a	42.23 ± 3.85 b	93.33 ± 6.67 a
PC [day]	4.68 ± 0.11 a	3.26 ± 0.12 b	3.12 ± 0.16 b
*A. montanum*			
GP [%]	63.33 ± 2.88 b	46.67 ± 5.77 c	73.33 ± 2.88 a
PC [day]	6.86 ± 0.29 a	1.04 ± 0.07 c	4.12 ± 1.12 b

* Different letters indicate means ± SD that are significantly different at α = 0.05 according to one-way ANOVA and post hoc Fisher’s test.

**Table 3:** The effects of different media compositions on the regeneration of shoots and roots in *Alyssum murale* after 12 weeks of in vitro cultivation as well as scores given in brackets for individual tested features depending on their intensity, the sum of which allowed the selection of optimal growth medium.

Medium Code	MC	Shoot Length [mm]	Rooted Explants [%]	Rooting Intensity *	Total Scores
A1	1.77 ± 0.87 c ** (1)	36.64 ± 9.50 a (3)	46.67 ± 5.77 c (2)	+ (1)	7
A2	1.62 ± 0.76 c (1)	20.30 ± 8.85 bc (1.5)	71.66 ± 2.88 b (3)	++ (2)	7.5
A3	3.13 ± 2.03 b (2)	25.66 ± 13.05 b (2)	25.00 ± 5.00 d (1)	+ (1)	6
A4	2.53 ± 0.51 b (2)	28.18 ± 13.12 b (2)	95.00 ± 5.00 a (4)	+++ (3)	11
A5	3.20 ± 0.84 b (2)	39.90 ± 12.14 a (3)	64.33 ± 4.04 b (3)	++ (2)	10
A6	4.83 ± 2.04 a (3)	14.76 ± 8.22 c (1)	55.00 ± 5.00 c (2)	+ (1)	6

* + spontaneous regeneration of up to 10 roots per explant, ++ more than 10 but less than 30 roots, +++ more than 30 roots. ** Different letters indicate means ± SD that are significantly different at α = 0.05 according to one-way ANOVA and post hoc Fisher’s test with the exception of shoot length when Tukey’s test for different N was applied.

**Table 4:** The effects of different media compositions on the regeneration of shoots and roots in *Alyssum alyssoides* after 12 weeks of in vitro cultivation as well as scores given in brackets for individual tested features depending on their intensity, the sum of which allowed the selection of optimal growth medium.

Medium Code	MC	Shoot Length [mm]	Rooted Explants [%]	Rooting Intensity *	Total Scores
A1	3.50 ± 0.85 b ** (2)	18.15 ± 6.66 a (2)	64.35 ± 4.04 b (3)	+ (1)	8
A2	2.95 ± 0.75 bc (1.5)	12.59 ± 2.95 b (1)	52.67 ± 3.06 c (2)	+ (1)	5.5
A3	2.81 ± 1.11 bc (1.5)	18.22 ± 5.06 a (2)	73.33 ± 2.89 a (4)	++ (2)	9.5
A4	5.00 ± 0.51 a (3)	14.46 ± 3.82 ab (1.5)	76.67 ± 2.89 a (4)	+++ (3)	11.5
A5	2.43 ± 1.13 c (1)	12.99 ± 2.31 ab (1.5)	34.33 ± 4.04 d (1)	+ (1)	4.5
A6	3.71 ± 0.95 b (2)	12.19 ± 2.31 b (1)	46.66 ± 7.64 c (2)	++ (2)	7

* + spontaneous regeneration of up to 10 roots per explant, ++ more than 10 but less than 30 roots, +++ more than 30 roots. ** Different letters indicate means ± SD that are significantly different at α = 0.05 according to one-way ANOVA and post hoc Fisher’s test with the exception of shoot length when Tukey’s test for different N was applied.

**Table 5:** The effects of different media compositions on the regeneration of shoots and roots in *Alyssum montanum* after 12 weeks of in vitro cultivation as well as scores given in brackets for individual tested features depending on their intensity, the sum of which allowed the selection of optimal growth medium.

Medium Code	MC	Shoot Length [mm]	Rooted Explants [%]	Rooting Intensity *	Total Scores
A1	2.86 ± 0.65 b ** (2)	32.89 ± 9.15 a (3)	70.00 ± 5.00 b (3)	+ (1)	9
A2	1.31 ± 0.48 c (1)	11.65 ± 2.34 c (1)	72.67 ± 2.52 b (3)	+ (1)	6
A3	1.63 ± 1.19 c (1)	9.87 ±3.64 c(1)	45.00 ± 5.00 c (2)	++ (2)	6
A4	1.31 ± 0.48 c (1)	23.75 ± 4.79 b (2)	70.67 ± 4.04 b (3)	+++ (3)	10
A5	4.29 ± 1.80 a (3)	13.56 ± 4.36 c (1)	15.00 ± 5.00 d (1)	+ (1)	6
A6	2.88 ± 1.56 b (2)	12.84 ±3.09 c (1)	85.00 ± 5.00 a (4)	+++ (3)	10

* + spontaneous regeneration of up to 10 roots per explant, ++ more than 10 but less than 30 roots, +++ more than 30 roots. ** Different letters indicate means ± SD that are significantly different at α = 0.05 according to one-way ANOVA and post hoc Fisher’s test with the exception of shoot length when Tukey’s test for different N was applied.

**Table 6:** The composition of tested media (A1–A6) based on WPM or MS salts and vitamins with some organic component modifications in relation to the original media formulation, enriched with different combinations of auxins (NAA, IAA) and cytokinins (2iP, BAP).

Medium Code	Basal Medium	Organic Component Additives	Plant Growth Regulators
A1	WPM	500 mg/L polyvinylpyrrolidone 500 mg/L myo-inositol 650 mg/L calcium gluconate 600 mg/L activated charcoal	2.5 mg/L 2iP * 1.0 mg/L NAA
A2	WPM	500 mg/L polyvinylpyrrolidone 500 mg/L myo-inositol 650 mg/L calcium gluconate	0.5 mg/L 2iP 0.1 mg/L IAA
A3	WPM	500 mg/L polyvinylpyrrolidone 100 mg/L myo-inositol 650 mg/L calcium gluconate	0.25 mg/L BAP 0.1 mg/L NAA
A4	MS	500 mg/L polyvinylpyrrolidone 100 mg/L myo-inositol 650 mg/L calcium gluconate	0.5 mg/L 2iP 0.1 mg/L IAA
A5	MS	100 mg/L myo-inositol 1.0 mg/L thiamine	0.25 mg/L BAP 0.1 mg/L NAA
A6	MS	100 mg/L myo-inositol 1.0 mg/L thiamine	0.5 mg/L BAP 0.1 mg/L NAA

* NAA: naphthaleneacetic acid; IAA: indole-3-acetic acid; 2iP: 6-(γ,γ-dimethylallylamino)purine; BAP: 6-benzyloaminopurine.

## Data Availability

The original contributions presented in the study are included in the article/Supplementary Materials, further inquiries can be directed to the corresponding author.

## Supplementary Materials

The following supporting information can be downloaded at: https://www.mdpi.com/article/10.3390/plants13223122/s1, Figure S1: Culture growth of different Alyssum species on media with the highest and lowest number of points given for the individual characteristics assessed.

## References

[1] Niazian M., Niedbała G. (2020). Machine Learning for Plant Breeding and Biotechnology. Agriculture.

[2] Bansal M., Mujib A., Bansal Y., Dewir Y.H., Mendler-Drienyovszki N. (2024). An Efficient In Vitro Shoot Organogenesis and Comparative GC-MS Metabolite Profiling of *Gaillardia pulchella* Foug. Horticulturae.

[3] Hasnain A., Naqvi S.A.H., Ayesha S.I., Khalid F., Ellahi M., Iqbal S., Hassan M.Z., Abbas A., Adamski R., Markowska D. (2022). Plants in vitro propagation with its applications in food, pharmaceuticals and cosmetic industries; current scenario and future approaches. Front. Plant Sci..

[4] Abdalla N., El-Ramady H., Seliem M.K., El-Mahrouk M.E., Taha N., Bayoumi Y., Shalaby T.A., Dobránszki J. (2022). An Academic and Technical Overview on Plant Micropropagation Challenges. Horticulturae.

[5] Muszyńska E., Hanus-Fajerska E., Koźmińska A. (2018). Differential Tolerance to Lead and Cadmium of Micropropagated *Gypsophila fastigiata* Ecotype. Water Air Soil Pollut..

[6] Sychta K., Słomka A., Sliwinska E., Migdałek G., Kuta E. (2020). From cells highly tolerant to Zn and Pb to fully fertile plants—Selection of tolerant lines with in vitro culture. Plant Physiol. Biochem..

[7] Muszyńska E., Hanus-Fajerska E., Piwowarczyk B., Augustynowicz J., Ciarkowska K., Czech T. (2017). From laboratory to field studies—The assessment of *Biscutella laevigata* suitability to biological reclamation of areas contaminated with lead and cadmium. Ecotoxicol. Environ. Saf..

[8] Hanus-Fajerska E., Ciarkowska K., Muszyńska E. (2019). Long-term field study on stabilization of contaminated wastes by growing clonally reproduced *Silene vulgaris* calamine ecotype. Plant Soil.

[9] Wijerathna-Yapa A., Hiti-Bandaralage J. (2023). Tissue Culture—A Sustainable Approach to Explore Plant Stresses. Life.

[10] Di Lonardo S., Capuana M., Arnetoli M., Gabbrielli R., Gonnelli C. (2011). Exploring the metal phytoremediation potential of three *Populus alba* L. clones using an in vitro screening. Environ. Sci. Pollut. Res. Int..

[11] Cano-Ruiz J., Ruiz Galea M., Amorós M.C., Alonso J., Mauri P.V., Lobo M.C. (2020). Assessing *Arundo donax* L. in vitro-tolerance for phytoremediation purposes. Chemosphere.

[12] Lin Q., Hamid Y., Yang H., Jiang J., Shan A., Wang M., Hussain B., Feng Y., Li T., He Z. (2023). Cadmium mobility and health risk assessment in the soil-rice-human system using in vitro biaccessibility and in vivo bioavailability assay: Two year field experiment. Sci. Total Environ..

[13] Muszyńska E., Hanus-Fajerska E., Ciarkowska K., Szarek-Łukaszewska G. (2020). Phytoremediation as an antidote to environmental pollution. Buckler Mustard (Biscutella laevigata L.) an Extraordinary Plant on Ordinary Mine Heaps Near Olkusz.

[14] Soleimani S.H., Bernard F., Amini M., Khavari-Nezhad R. (2020). Cadmium accumulation and alkaloid production of *Narcissus tazetta* plants grown under in vitro condition with cadmium stress. Plant Physiol. Rep..

[15] Płażek A., Dubert F. (2022). In Vitro Culture as a Tool for Studying Plant Developmental Processes at the Physiological Level in Poland. Acta Soc. Bot. Pol..

[16] Kwiatkowska M., Kłosowska K., Kurczyńska E.U. (2021). Germline development and seed set of metallophyte *Biscutella laevigata* L. (Brassicaceae). Flora.

[17] Ruiz-Huerta E.A., Armienta-Hernández M.A., Dubrovsky J.G., Gómez-Bernal J.M. (2022). Bioaccumulation of heavy metals and As in maize (*Zea mays* L) grown close to mine tailings strongly impacts plant development. Ecotoxicology.

[18] van der Ent A., Baker A.J.M., Reeves R.D., Pollard A.J., Schat H. (2013). Hyperaccumulators of metal and metalloid. Trace elements: Facts and fiction. Plant Soil.

[19] Broadhurst C.L., Chaney R.L. (2016). Growth and Metal Accumulation of an *Alyssum murale* Nickel Hyperaccumulator Ecotype Co-cropped with *Alyssum montanum* and Perennial Ryegrass in Serpentine Soil. Front. Plant Sci..

[20] Küpper H., Lombi E., Zhao F.J., Wieshammer G., McGrath S.P. (2001). Cellular compartmentation of nickel in the hyperaccumulators *Alyssum lesbiacum*, *Alyssum bertolonii* and *Thlaspi goesingense*. J. Exp. Bot..

[21] Feigl G., Varga V., Molnár Á., Dimitrakopoulos P.G., Kolbert Z. (2020). Different Nitro-Oxidative Response of *Odontarrhena lesbiaca* Plants from Geographically Separated Habitats to Excess Nickel. Antioxidants.

[22] Liu Z., Zhou L., Gan C., Hu L., Pang B., Zuo D., Wang G., Wang H., Liu Y. (2023). Transcriptomic analysis reveals key genes and pathways corresponding to Cd and Pb in the hyperaccumulator *Arabis paniculata*. Ecotoxicol. Environ. Saf..

[23] Sari S.H.J., Chien M.F., Inoue C. (2023). Cadmium and zinc accumulation behaviour of hyperaccumulator *Arabidopsis halleri* ssp. *gemmifera* in the hydroponic system. J. Degrad. Min. Lands Manag..

[24] Drozdova I.V., Alekseeva-Popova N.V., Kalimova I.B., Belyaeva A.I., Smirnova N.A. (2017). The accumulating ability and nickel tolerance of Brassicaceae species of the North Caucasus in connection with the problem of phytoremediation. J. Geochem. Explor..

[25] Muszyńska E., Labudda M., Hanus-Fajerska E. (2019). Changes in proteolytic activity and protein carbonylation in shoots of *Alyssum montanum* ecotypes under multi-metal stress. J. Plant Physiol..

[26] Labudda M., Dziurka K., Fidler J., Gietler M., Rybarczyk-Płońska A., Nykiel M., Prabucka B., Morkunas I., Muszyńska E. (2022). The Alleviation of Metal Stress Nuisance for Plants—A Review of Promising Solutions in the Face of Environmental Challenges. Plants.

[27] Sarath G., Bethke P.C., Jones R., Baird L.M., Hou G., Mitchell R.B. (2006). Nitric oxide accelerates seed germination in warm-season grasses. Planta.

[28] Kara Z., Yazar K., Doğan O., Vergili E. (2020). Sodium Nitroprusside and Gibberellin Effects on Seed Germination and Seedling Development of Grapevine (*Vitis vinifera* L.) Cvs. Ekşi Kara and Gök Üzüm. Erwerbs-Obstbau.

[29] Chu C., Poore R.C., Bolton M.D., Fugate K.K. (2020). Mechanism of Sugarbeet Seed Germination Enhanced by Hydrogen Peroxide. Front. Plant Sci..

[30] Habib N., Ali Q., Ali S., Haider M.Z., Javed M.T., Khalid M., Perveen R., Alsahli A.A., Alyemeni M.N. (2021). Seed Priming with Sodium Nitroprusside and H_2_O_2_ Confers Better Yield in Wheat Under Salinity: Water Relations, Antioxidative Defense Mechanism and Ion Homeostasis. J. Plant Growth Regul..

[31] dos Santos Guaraldo M.M., Pereira T.M., dos Santos H.O., de Oliveira T.L., Pereira W.V.S., Von Pinho E.V.d.R. (2023). Priming with sodium nitroprusside and hydrogen peroxide increases cotton seed tolerance to salinity and water deficit during seed germination and seedling development. Environ. Exp. Bot..

[32] Silva V.N., Bernardes M.M., Pereira A.A.S., Ferreira R.A., Pereira E.G., Bicalho E.M. (2023). Seed Priming of *Handroanthus heptaphyllus* for the Restoration of the Mining Fields. Water Air Soil Pollut..

[33] Mañas P., De las Heras J. (2018). Phytotoxicity test applied to sewage sludge using *Lactuca sativa* L. and *Lepidium sativum* L. seeds. Int. J. Environ. Sci. Technol..

[34] Bethke P.C., Gubler F., Jacobsen J.V., Jones R.L. (2004). Dormancy of *Arabidopsis* seeds and barley grains can be broken by nitric oxide. Plant.

[35] Zhu Z.H., Sami A., Xu Q.Q., Wu L.L., Zheng W.Y., Chen Z.P., Jin X.Z., Zhang H., Li Y., Yu Y. (2021). Effects of seed priming treatments on the germination and development of two rapeseed (*Brassica napus* L.) varieties under the co-influence of low temperature and drought. PLoS ONE.

[36] Bailly C., El-Maarouf-Bouteau H., Corbineau F. (2008). From intracellular signaling networks to cell death: The dual role of reactive oxygen species in seed physiology. Comptes Rendus Biol..

[37] Šírová J., Sedlářová M., Piterková J., Luhová L., Petřivalský M. (2011). The role of nitric oxide in the germination of plant seeds and pollen. Plant Sci..

[38] Barba-Espín G., Diaz-Vivancos P., Job D., Belghazi M., Job C., Hernández J.A. (2011). Understanding the role of H_2_O_2_ during pea seed germination: A combined proteomic and hormone profiling approach. Plant Cell Environ..

[39] Wiszniewska A., Labudda M., Muszyńska E. (2023). Response to Cadmium in *Silene vulgaris* Ecotypes Is Distinctly Affected by Priming-Induced Changes in Oxidation Status of Macromolecules. Int. J. Mol. Sci..

[40] Babaoğlu Aydaş S.S., Açik L., Leduc D., Adigüzel N., Ellialtioğlu S.S., Suludere Z., Kadioğlu Y.K. (2013). Localization and distribution of nickel and other elements in in-vitro grown *Alyssum corsicum* exhibiting morphological changes in trichomes: Initial insights into molecular mechanisms of nickel hyperaccumulation. Turk. J. Bot..

[41] Muszyńska E., Hanus-Fajerska E. (2017). In vitro multiplication of *Dianthus carthusianorum* calamine ecotype with the aim to revegetate and stabilize polluted wastes. Plant Cell Tissue Organ Cult..

[42] Shukla S.P., Khare P.B. (2014). In vitro conservation of some threatened and economically important ferns belonging to the Indian subcontinent. J. Bot..

[43] Xu J., Zhang Y.X., Chai T.Y., Guan Z.Q., Wei W., Han L., Cong L. (2008). In vitro multiplication of heavy metals hyperaccumulator *Thlaspi caerulescens*. Biol. Plant..

[44] Corso G.D., Borgato L., Furini A. (2005). In vitro plant regeneration of the heavy metal tolerant and hyperaccumulator *Arabidopsis halleri* (Brassicaceae). Plant Cell Tissue Organ Cult..

[45] Jack E.M., Anatasova S., Verkleij J.A.C. (2005). Callus induction and plant regeneration in the metallophyte *Silene vulgaris* (Caryophyllaceae). Plant Cell Tissue Organ Cult..

[46] Zhao S.J., Zhang Z.C., Gao X., Tohsun G., Qiu B.S. (2009). Plant regeneration of the mining ecotype *Sedum alfredii* and cadmium hyperaccumulation in regenerated plants. Plant Cell Tissue Organ Cult..

[47] Ivanović S., Marković M., Milutinović M., Skočajić D. (2022). In vitro propagation of *Dianthus cruentus* and acclimatization in hydroponic culture. Phyton.

[48] Surya M.I., Ismaini L., Lailaty I.Q. (2019). In vitro regeneration and induction of mutation in loquat (*Eriobotrya japonica* L.). Aust. J. Crop Sci..

[49] Muszyńska E., Tokarz K., Dziurka M., Labudda M., Dziurka K., Piwowarczyk B. (2021). Photosynthetic apparatus efficiency, phenolic acid profiling and pattern of chosen phytohormones in metal-tolerant and intolerant *Alyssum montanum* ecotypes. Sci. Rep..

[50] Cetlová V., Zozomová-Lihová J., Melichárková A., Mártonfiová L., Španiel S. (2021). Multiple Drivers of High Species Diversity and Endemism Among Alyssum Annuals in the Mediterranean: The Evolutionary Significance of the Aegean Hotspot. Front. Plant Sci..

[51] Muszyńska E., Labudda M., Kral A. (2020). Ecotype-Specific Pathways of Reactive Oxygen Species Deactivation in Facultative Metallophyte *Silene vulgaris* (Moench) Garcke Treated with Heavy Metals. Antioxidants.

[52] Purmale L., Jēkabsone A., Andersone-Ozola U., Karlsons A., Osvalde A., Ievinsh G. (2022). Comparison of In Vitro and *In Planta* Heavy Metal Tolerance and Accumulation Potential of Different *Armeria maritima* Accessions from a Dry Coastal Meadow. Plants.

[53] Fourati E., Vogel-Mikuš K., Bettaieb T., Kavčič A., Kelemen M., Vavpetič P., Pelicon P., Abdelly C., Ghnaya T. (2019). Physiological response and mineral elements accumulation pattern in *Sesuvium portulacastrum* L. subjected in vitro to nickel. Chemosphere.

[54] Nouri M., Haddioui A. (2021). Improving seed germination and seedling growth of *Lepidium sativum* with different priming methods under arsenic stress. Acta Ecol. Sin..

[55] Murashige T., Skoog F. (1962). A revised medium for rapid growth and bioassays with tobacco tissue culture. Physiol. Plant..

[56] Rochalska M., Orzeszko-Rywka A., Seroka J., Najgrodzka A. (2015). Priming of red beet and sugar beet seed using the infusions of chamomile and sage. J. Res. Appl. Agric. Eng..

[57] Lloyd G., McCown B. (1980). Commercially-feasible micropropagation of mountain laurel, *Kalmia latifolia*, by use of shoot-tip culture. Comb. Proc. Int. Plant Propagators’ Soc..

