# Supplementary material for: In Vitro Propagation of Alyssum Species with Different Metal Accumulation Strategies
Source: Plants (Basel). 2024 Nov 6;13(22):3122. doi: 10.3390/plants13223122 (PMC11597741; doi:10.3390/plants13223122)
Supplement: Supplementary file 1 [file plants-13-03122-s001.zip › plants-3258221-supplementary.pdf]

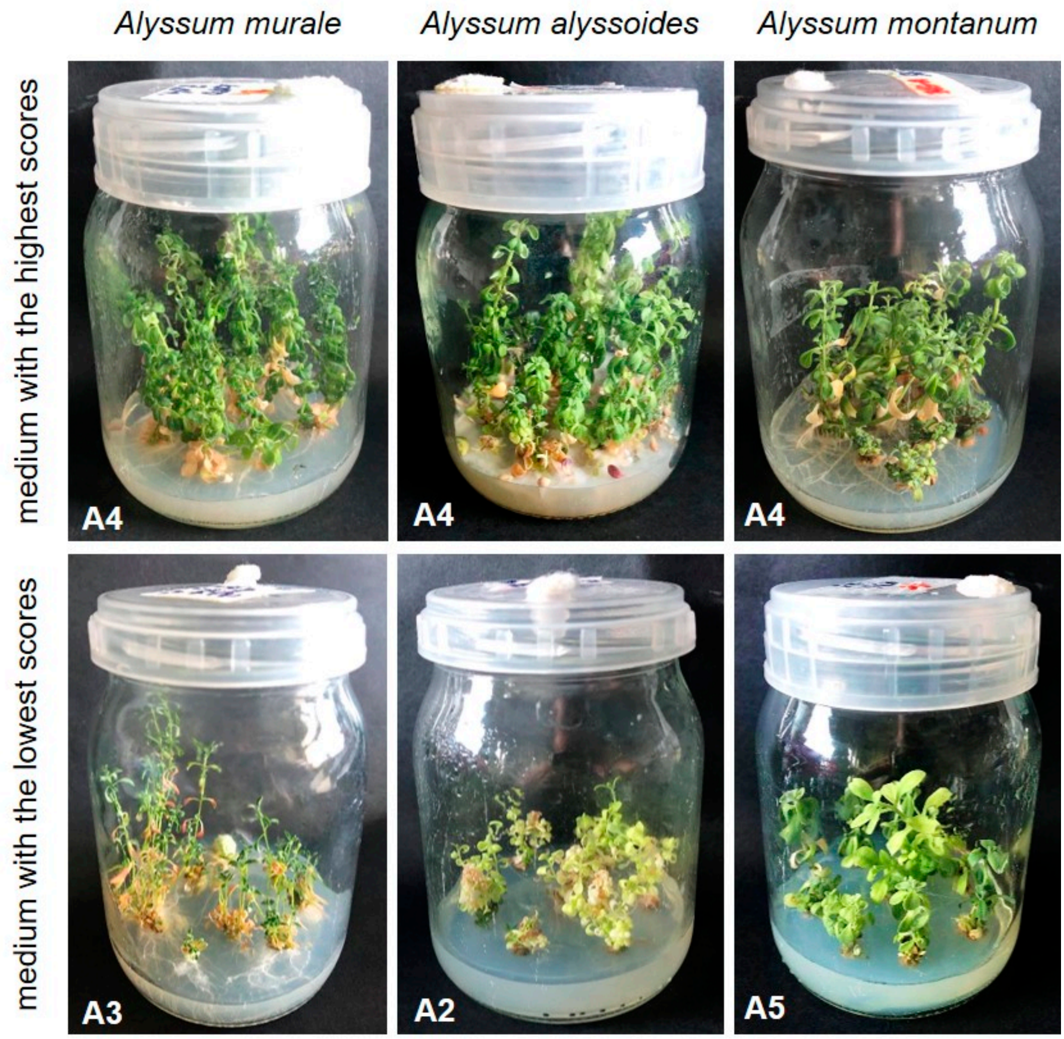

Figure S1. Culture growth of different Alyssum species on media with the highest and lowest number of points given for the individual characteristics assessed.
